# Application of Convolutional Neural Networks for Determining Gender and Age in Forensic Dentistry

**DOI:** 10.7759/cureus.73028

**Published:** 2024-11-05

**Authors:** Madzida Hundur Hiyari, Mirza Pasic, Selma Zukic

**Affiliations:** 1 Artificial Intelligence, Verlab Research Institute for Biomedical Engineering, Medical Devices and Artificial Intelligence, Sarajevo, BIH; 2 Industrial Engineering and Management, University of Sarajevo - Faculty of Mechanical Engineering, Sarajevo, BIH; 3 Dental Morphology, University of Sarajevo - Faculty of Dentistry With Dental Clinical Center, Sarajevo, BIH

**Keywords:** artificial intelligence in medicine, big data analytics and machine learning, dental clinic, forensic dentistry, orthopantomogram (opg)

## Abstract

Background

Determining human identity has always been important in forensic investigations. Forensic dentistry has developed significantly having a key role in determining gender and age. One of the methods that is important in forensic dentistry is the analysis of orthopantomograms, which are X-rays of the complete upper and lower jaw, including the surrounding anatomical structures. The uniqueness of the dental features recorded in orthopantomograms makes them useful for individual identification, more specifically for the assessment of gender and age. This study was conducted to evaluate the application of convolutional neural networks in automating the process of gender and age estimation based on orthopantomograms, to improve accuracy and efficiency in forensic dentistry.

Methodology

Convolutional neural networks are powerful tools in the field of artificial intelligence for image processing and analysis because their convolutional layers extract specific features that are characteristic of a certain class. A total of 3716 orthopantomograms collected from the database of the University of Sarajevo - Faculty of Dentistry with the Dental Clinical Center were used to create convolutional neural network models for predicting gender and age. The orthopantomograms were taken in the period from January to December 2022 for the needs of doctors and providing services to patients at four polyclinics: Clinic for Dental Diseases and Endodontics, Clinic for Oral Diseases and Periodontology, Clinic for Oral Surgery, and Clinic for Pediatric and Preventive Dentistry.

Results

The results derived from three developed models confirm that the developed convolutional neural networks have high accuracy. The first model estimated gender, while the second and the third models estimated age within certain age ranges, the second from 12 to 24 years, and the third from 20 to 70 years. After training on the training dataset, all models achieved high accuracy on the validation dataset. The models demonstrated high accuracy without signs of overfitting, with the first model achieving 95.98%, the second model achieving 97.90%, and the third model achieving 96.12% accuracy.

Conclusion

This research concluded that the developed convolutional neural networks for gender and age estimation from orthopantomograms showed high accuracy. Models' predictions of gender and two age groups exceeded 95% accuracy. Therefore, convolutional neural networks can be considered useful tools for gender and age determination in forensic dentistry and can facilitate and speed up the processes of assessment and determination of essential characteristics.

## Introduction

Forensic dentistry is an important discipline in forensic science for gender and age determination in legal and criminal investigations through dental records. Using artificial intelligence (AI) in forensic dentistry by providing tools for analyzing dental traces and helping in age and gender determination of individuals is in big expansion. Artificial neural networks (ANNs), specifically convolutional neural networks (CNNs) are one of the methods of AI that are being widely used in image classification problems. Because CNNs have proven to be a powerful tool for image classification and recognition of hidden features within images, they have also found their application in forensic dentistry.

Artificial intelligence in dentistry

In research [1] CNNs showed high accuracy comparable to that of a board-certified periodontist for classifying dental implant systems using panoramic and periapical radiographic images. AI is being used in in various subfields of dentistry, including periodontology, endodontics, orthodontics, prosthodontics, and oral pathology [2]. This review paper shows how using AI can improve disease diagnosis, treatment planning, and risk assessment, despite certain limitations such as data standardization and integration into clinical workflows. The ability of AI to enhance diagnosis is further validated in the field of endodontics, where AI models have shown efficacy in diagnosing apical lesions, detecting dental caries, and aiding in treatment planning [3]. The application of ANNs and CNNs in dental diagnostics is further researched in [4] and [5], where classifying different types using ANNs was examined. In [4], ANNs were able to accurately classify patients into aggressive or chronic periodontitis groups based on immunological datasets showing potential for AI to distinguish different dental features that are being used for forensic purposes. In [5] an overview of AI in prosthodontics, including diagnostic and predictive applications is shown highlighting the increasing usage of AI methods in dental practice. Using AI in orthodontics has also been investigated in [6], where CNNs and ANNs were used from automated anatomic landmark detection to orthodontic treatment planning. The study shows that AI methods such as CNNs and ANNs achieved precision and accuracy comparable to that of orthodontic specialists. Using AI in dental radiography from the diagnosis of dental caries and periodontal disease to forensic odontology and implant recognition is analyzed in [7]. CNNs can show improvement in accuracy and efficiency, given that dental radiographs are a critical tool in forensic identification. In [8], a survey was conducted among dental residents and dentists in Navi Mumbai. Out of all participants, 68% answered that AI aids in improving diagnostic procedures and treatment planning, while 65% of them will likely adopt AI in their practices in the next five years [8]. A review [9] analyzed the awareness of different groups towards AI. This review study analyzed 7,688 participants across 21 cross-sectional studies. The awareness varied between different groups with the lowest awareness being among the dental students group [9]. The findings of this review highlight the current awareness and potential for adoption as well as the bridge between AI integration and dental education. The use of artificial intelligence in patient education and communication has facilitated the use of personalized patient education, virtual consultation, and translation services, although many problems can arise from the use of too much artificial intelligence in patient education and communication, such as over-reliance and privacy concerns [10]. Robotic technology and AI have improved precision and efficiency in implant dentistry by reducing human error. The integration of artificial intelligence with robotic technology has improved patient data analysis, diagnosis, treatment planning, and implant design [11]. Furthermore, AI's role in endodontics has been explored, demonstrating its utility in studying root canal anatomy, predicting stem cell viability, identifying root fractures, and improving treatment accuracy. The analysis of AI models in terms of cost-effectiveness and applicability remains [12]. A bibliometric analysis looked at the use of artificial intelligence in the dental literature from 2011 to 2021. The analysis revealed significant growth in publications related to artificial intelligence in the fields of radiology, orthodontics, and general diagnostic methods [13]. A three-step algorithm for gender estimation from dental X-ray images was done at universities in India. The first step involved image preprocessing, the second step was segmentation, and the final step was the classification of images by gender. A database of 1000 dental images, divided into 600 images for training and 400 for testing, resulted in an achieved gender estimation accuracy of 98.27% [14]. As part of research conducted at universities in Zagreb and Split, it was proposed to use an automated solution for gender estimation based on deep learning, utilizing convolutional neural networks. The dataset analyzed included 4000 panoramic dental X-ray images of patients of European origin, captured with a wide range of orthopantomograms (OPGs). The method enabled the assessment of 64 images per second on modern hardware, without human intervention, achieving an exceptionally high accuracy of 96.87% ± 0.96% [15]. In the research [16], the team of researchers conducted four additional experiments to determine whether specific anatomical structures visible in X-ray images contribute to gender estimation. Based on a database of 4155 X-ray images, the accuracy of gender estimation was 94.3% [16]. In [17] gender and age were assessed by using three different image preprocessing methods. The second method, where the input image was first converted to grayscale and then the Foreground Enhance method was applied in combination with the similarity rate, proved to be the most successful in age estimation. Research conducted at universities in China is one of the few studies on the topic of age estimation using convolutional neural networks based on a large number of orthopantomograms. The study was conducted on a database of 10,257 orthopantomograms. A logistic regression linear model was derived for each legal age threshold (14, 16, and 18 years), as well as an end-to-end convolutional neural network that directly classified age for comparison with the manual method. Both methods were based on the left mandibular third molar. The results showed that compared to the accuracy of the manual method of 92.5%, 91.3%, and 91.8% for legal age thresholds of 14, 16, and 18 years, respectively, the convolutional neural network provided a more accurate estimation with results of 95.9%, 95.4%, and 92.3% accuracy [18]. Research to determine the age of children based on OPG images using CNNs was conducted in Malaysia with children aged between 1 and 17 years. This research was applied on 456 images and CNNs showed high accuracy and precision in age estimation. Age estimation for male children proved to be simpler, with the highest accuracy achieved for children between 14 and 17 years old [19]. Research [20] focused solely on orthopantomograms of good radiological quality, without any conditional dental characteristics. Two fully automatic methods for age estimation based on OPGs were presented in the study. The first method, DANet, used a sequential convolutional neural network to predict age, while the second method, DASNet, used a different convolutional neural network pathway for age prediction, including gender-specific features to improve age prediction accuracy. Both methods were tested on a dataset of 2289 images of persons aged 4.5 to 89.2 years [20]. The results showed that DASNet was more effective than DANet with a lower mean error and mean absolute error. Analysis [21] over 2000 X-ray images were divided into seven classes ranging from 2 to 90 years of age for training various convolutional neural network architectures. However, due to factors such as tilt, overlap, and the absence of teeth, the research showed a low accuracy of less than 40% in using dental images for age estimation using developed convolutional architectures. In addition to well-known architectures, the study also proposed the CapsuleNet architecture for age estimation from dental images, which showed a 36% improvement over previous convolutional neural network architectures and transfer learning, achieving an overall accuracy of 76% [21]. Forensic odontology serves as a crucial methodology in the identification of individuals through the comparison of antemortem and postmortem dental records particularly of panoramic radiographs (PRs) which showed reliable identification of unknown individuals [22]. An approach was proposed to improve and automate the identification process by applying computer vision techniques to compare antemortem and postmortem PRs with a success rate of 85%. Review research [23] investigated the application of AI in forensic odontology which included the identification of bite marks, mandibular morphology prediction, gender determination, and age estimation. ANN and CNN models showed high accuracy and precision that was comparable to trained forensic specialists. The importance of dental tissues in forensic identification is underlined by the durability of teeth and the resilience of dental restoration materials even under extreme conditions, such as acid exposure. A novel approach of AI algorithms to the 3D reconstruction of dental patterns was introduced by [24], involving advanced imaging technologies including micro-computed tomography, cone-beam computed tomography (CBCT), and Fourier transform infrared spectroscopy combined with AI algorithms. In [25], intraoral scanner images were used to classify individual molar teeth based on metallic restorations using a pre-trained AI model with transfer learning. The algorithm achieved an accuracy of 95% showing its potential for efficiently generating dental charts and contributing to faster identification in postmortem scenarios particularly after mass disasters. Paired OPGs obtained at different times were analyzed using CNNs for identification purposes [26]. The study showed the efficacy of deep learning methods for personal identification using limited numbers of orthopantomographic images with fine-tuning pre-trained models having the highest identification accuracy. In an umbrella review [27], the quality of systematic reviews was analyzed with a focus on gender determination using dental procedures, such as evaluating the most dimorphic teeth and analyzing oral tissue remnants for DNA. Despite the potential utility of these methods, the quality of evidence was generally rated as low, indicating a need for better-designed clinical trials and systematic reviews to validate these procedures.

Convolutional neural networks

CNNs are a type of ANN created for processing and analyzing data with spatial patterns, such as images and videos [28]. CNNs consist of convolutional and pooling layers that have the ability to identify hidden features from data [29]. Using gradient-based learning, CNNs adjust their internal parameters to optimize feature representation and reduce the error between predicted and actual values. With deep learning advancements, CNNs have emerged as essential tools in visual data processing, especially in image feature recognition. CNNs build a feature hierarchy by layering simple features to form complex representations, accomplished through a series of convolutional, pooling, and fully connected layers. Fully connected layers further process this data, creating hidden layers and an output layer as in ANNs where the final prediction is done [30].

The aim of this research was to develop convolutional neural network models for gender and age prediction based on dental X-ray images (orthopantomograms) with high accuracy. In this research, CNNs were used to identify features that could differentiate between males and females, as well as classify individuals into specific age groups. The contribution of this research lies in the development of convolutional neural network models for age and gender estimation. This research not only provides high-accuracy models but also establishes a methodological framework that can be applied or adapted for similar forensic identification tasks.

## Materials and methods

Materials and methods are discussed in terms of data collection and the development of CNN models for gender and age estimation.

Data collection 

The input data used in this research and application of convolutional neural networks for gender and age estimation in forensic odontology were orthopantomograms. A total of 3,716 orthopantograms of known gender and age collected at the Faculty of Dentistry of the University of Sarajevo were used. Orthopantomograms were collected from the University of Sarajevo - Faculty of Dentistry with the Dental Clinical Center. The collected orthopantomograms were taken between January and December 2022 for the use of doctors and patient services at four clinics: Clinic for Dental Diseases and Endodontics, Clinic for Oral Diseases and Periodontology, Clinic for Oral Surgery, and Clinic for Pediatric and Preventive Dentistry. All orthopantomograms (X-ray images) were acquired with Orthophos SL 2D device (Sirona Dental Systems GmbH, Germany) with 60-90 kV and 3-16 mA.

Certain steps were taken and criteria were set for inclusion or exclusion of images from the analysis in order to ensure the quality of orthopantomograms in the dataset used for this research. Special attention was paid to the quality of the orthopantomogram images and only high-quality images were included in the dataset for analysis to ensure efficiency and reduce the possibility of errors. It was carefully checked whether all relevant dental and bone parts were visible in the images. Images with visible technical deficiencies or poor visualization of anatomical structures were excluded to ensure the reliability and quality of the results. Images with visible bone fractures, deformities, injuries, loss of functional dentition, and/or dental implants and prosthetic restorations were excluded, as well as the lack of teeth in the jaw. According to the WHO (World Health Organization), functional dentition is defined as no less than 20 teeth throughout life with no need for tooth replacement.

Since gender and age estimation is based on the characteristics of dental structures, images without the presence of a significant number of teeth were not relevant to the research objectives; therefore, such images were excluded to ensure the highest possible accuracy of the results. Through the application of these criteria and procedures, the quality and standardization of the orthopantomograms in the dataset were ensured, and any possible variability was minimized.

The data were divided by gender which was determined based on patient names and by chronological age stored in electronic records. For the male class, 1700 orthopantomograms were collected, while for the female class, 2016 orthopantomograms were collected, which represented the input data of the first model, gender estimation. The ages of patients from the collected orthopantomograms were determined based on the year of birth which ranged from 1930 to 2022. The second and third developed models estimated age. The second model estimated ages from 12 to 24 years with three classes: younger than 16 years, from 17 to 18 years, and older than 18 years, and the third model estimated ages from 20 to 70 years in 5 classes, with a range of 10 years.

When collecting orthopantomograms for this research, all relevant ethical guidelines, standards, and regulations were strictly followed. All patients signed an agreement that all collected data may be used for scientific and research purposes with the approval of the ethics committee when taking orthopantomograms. Ethical approval was obtained from the relevant institutional ethics committee from the University of Sarajevo - Faculty of Dentistry with Dental Clinical Center, which is responsible for protecting the rights and welfare of individuals (approval number 01-4-2-1-9/2023). Certain measures were taken to ensure the anonymization and to protect the privacy of the individuals to whom the images belong. Technical measures were also implemented to provide the security and protection of the orthopantomograms from unauthorized access or misuse. Orthopantomograms were previously taken for diagnostic and clinical purposes. For this research, only archived images were used, with their use limited to the specified research objectives.

Development of CNN models for gender and age estimation

In this research, three CNN models were developed for gender estimation and age estimation using dental X-ray images (orthopantomogram). The first model was developed to determine gender, distinguishing between male and female patients, while the second and third models focused on estimating age within defined ranges. Model 2 classified individuals aged between 12 to 24 years into three categories, while Model 3 was developed to classify individuals from 20 to 70 years, categorizing individuals into five 10-year intervals. Model 2 was developed to test the usability in cases of determining legal adulthood. Therefore, three categories were created within that model: younger than 16, which corresponds to the limit of legal minors and younger adolescents in Bosnia and Herzegovina, 17-18, which corresponds to the limit of older adolescents, and older than 18, which corresponds to the limit of legal adulthood in Bosnia and Herzegovina. Model 3 was developed to test the use of CNNs in estimating the age of adults, so within that model, categories with an interval of 10 years were set. The goal of these models was to provide a tool for forensic investigations, where age estimation can aid significantly in narrowing down identification by providing a more precise age group, which is often challenging to determine using dental records alone.

All three models were CNNs architecture to find hidden features from dental X-ray images and then classify these images into different gender or age groups. Each model consists of convolutional layers followed by pooling layers for feature extraction and dimensionality reduction. After convolutional and pooling layers, Flatten layer converts 2d data into a one-dimensional vector, and then there is a fully connected (dense) layer for classification. The activation functions used for the convolutional and dense layers were ReLU, and sigmoid or a softmax activation functions were used in the output layer depending on the model. The images were preprocessed using ImageDataGenerator to rescale pixel values of images to a range of 0 to 1. Models were trained with a batch size of 16 and a different number of epochs.

The first model for gender estimation had the task of distinguishing between male and female subjects based on X-ray images of size 256x256 pixels. The architecture of the CNN used for the first model consisted of three convolutional layers with 16, 32, and 64 filters respectively, and with a kernel size of (3, 3) followed by a MaxPooling layer with a pool size of (2, 2). After these convolutional layers, a Flatten layer was used to convert the two-dimensional output into a one-dimensional vector. The Flatten layer was followed by a dense layer of 512 hidden neurons with a ReLU activation function. The output layer had one neuron with a sigmoid activation function that was used for binary classification between male and female subjects. Table 1 shows the structure of CNN used for the first model.

**Table 1:** Structure of CNN used for gender estimation model (model 1) CNN: convolutional neural network

Layer (type)	Output Shape
Conv2D	(254, 254, 16)
MaxPooling2D	(127, 127, 16)
Conv2d	(125, 125, 32)
MaxPooling2D	(62, 62, 32)
Conv2d	(60, 60, 64)
MaxPooling2D	(30, 30, 64)
Flatten	(57600)
Dense	(512)
Dense	(1)

The dataset for this model consisted of 3716 images. For the training set, 2471 images were used, while 1245 images were used for the validation set. All images were divided into two classes male and female and the model was trained for 15 epochs. The model was trained with the RMSprop optimizer with a learning rate of 0.001. Binary crossentropy was the loss function and accuracy was the evaluation metric.

The second model was used for age estimation in the range of 12 to 24 years by classifying individuals into three categories. The categories are under 16 years, 17 to 18 years, and over 18 years. This model used an input image size of 224x224 pixels and consisted of ten layers: three convolutional layers with 32, 64, and 128 filters, respectively, each with a kernel size of (3, 3) and followed by a MaxPooling layer with a pool size of (2, 2). After convolutional layers, a Flatten layer was used to convert the two-dimensional output into a one-dimensional vector followed by a dense layer of 128 hidden neurons with a ReLU activation function. The output layer had three neurons with a softmax activation function. Table 2 shows the structure of CNN used for the second model.

**Table 2:** Structure of CNN used for the age estimation model from 12 to 24 years (model 2) CNN: convolutional neural network

Layer (type)	Output Shape
Conv2D	(222, 222, 32)
MaxPooling2D	(111, 111, 32)
Conv2d	(109, 109, 64)
MaxPooling2D	(54, 54, 64)
Conv2d	(52, 52, 128)
MaxPooling2D	(26, 26, 128)
Flatten	(86528)
Dense	(128)
Dense	(3)

The dataset for this model consisted of 1193 images, where 716 images were used as the training set and 477 images were used as the validation set. The model was trained for 10 epochs and Adam optimizer was used. Categorical crossentropy was used as a loss function, while accuracy was used as the evaluation metric. All images were split into three classes younger than 16 years, between 17 and 18 years, and older than 18 years. 

The third model estimated age within a broader range from 20 to 70 years, categorizing individuals into five classes, each covering a 10-year interval. The model's structure was the same as the structure of Model 2. The only difference is in the output layer, where there were five neurons instead of three. Softmax activation function was used in the output layer. Table 3 shows the architecture of the CNN used for the third model.

**Table 3:** Structure of CNN used for the age estimation model from 20 to 70 years (model 3) CNN: convolutional neural network

Layer (type)	Output Shape
Conv2D	(222, 222, 32)
MaxPooling2D	(111, 111, 32)
Conv2d	(109, 109, 64)
MaxPooling2D	(54, 54, 64)
Conv2d	(52, 52, 128)
MaxPooling2D	(26, 26, 128)
Flatten	(86528)
Dense	(128)
Dense	(3)

The model was trained for 10 epochs using the Adam optimizer, categorical crossentropy as the loss function, and accuracy as the evaluation metric. The total number of pictures used for model 3 was 3020, where 1,835 images were used as the training set, and 1,185 images were used as the validation set. All images were categorized into five different classes, each covering a 10-year age range from 20 to 70 years.

All three CNN models provided specialized applications for forensic dentistry-Model 1 for binary gender classification, and Models 2 and 3 for age estimation across different age ranges. Each developed model used convolutional and pooling layers to recognize hidden features from X-ray images, followed by dense layers to classify these features accurately. Sigmoid and softmax activation functions were used for binary and multi-class classification respectively. Developed models have the potential to be used in real-world forensic investigations, especially for narrowing down identity by providing gender and age estimates. Accuracy was used as the evaluation metric for all three models in order to determine how each network classified the dental X-ray images according to gender or age groups as indicated in the formula below:

$accuracy=\frac{correct\;predictions}{all\;predictions}$

For the gender estimation model, accuracy provided a straightforward assessment of the model's ability to correctly differentiate between male and female subjects. For the age estimation models, accuracy evaluated the ability of the networks to correctly classify subjects into appropriate age categories. High accuracy in these models is important for classifying individuals into adequate gender and age groups which can significantly improve forensic investigations and reduce errors in the identification process.

## Results

The first model developed was specifically designed for gender estimation, providing valuable insights into identifying males and females based on their dental X-ray images. Furthermore, two additional models were developed for age estimation, considering specific age groups. The first model estimated the age from 12 to 24 years with three classes: younger than 16 years, from 17 to 18 years, and older than 18 years, and the second model made an assessment from 20 to 70 years of age in 5 classes, with a range of 10 years. Recognizing age ranges plays a crucial role in forensic dentistry as it allows narrowing down the identification range and provides valuable guidance in investigations. The reason for choosing specific age groups in the models is their applicability in real forensic investigations, as they offer precise estimates for dental age, which is harder to determine.

Gender estimation

**Figure 1:**
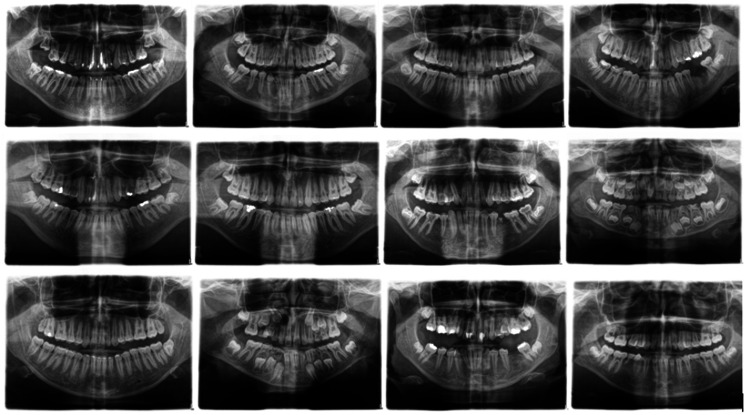
Sample of 12 images for gender estimation model (model 1)

The first developed model focused on estimating individuals' gender based on 3716 orthopantomograms, employing a convolutional neural network. Figure 1 shows a random sample of 12 out of a total of 3716 orthopantomograms used for the development of the model for gender estimation.

**Figure 2:**
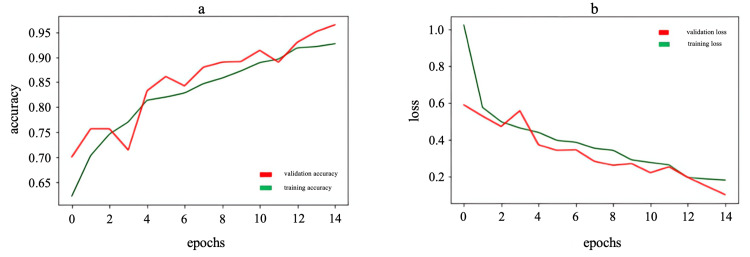
Accuracy (a) and loss (b) graphs for gender estimation model (model 1)

By analyzing the graphs in Figure 2, one can assess how the model performance changes over epochs and determine whether there is overfitting or other problems. The graphs show that accuracy increases and loss decreases during the training and validation of CNN models. These graphs are good because they show several key things: an increase in precision, a decrease in loss, convergence between the training and validation lines, and the absence of significant overfitting. An increase in precision indicates that the model is learning and getting better at classifying the data, while a decrease in loss means that the model is becoming more accurate and minimizing errors.

**Figure 3:**
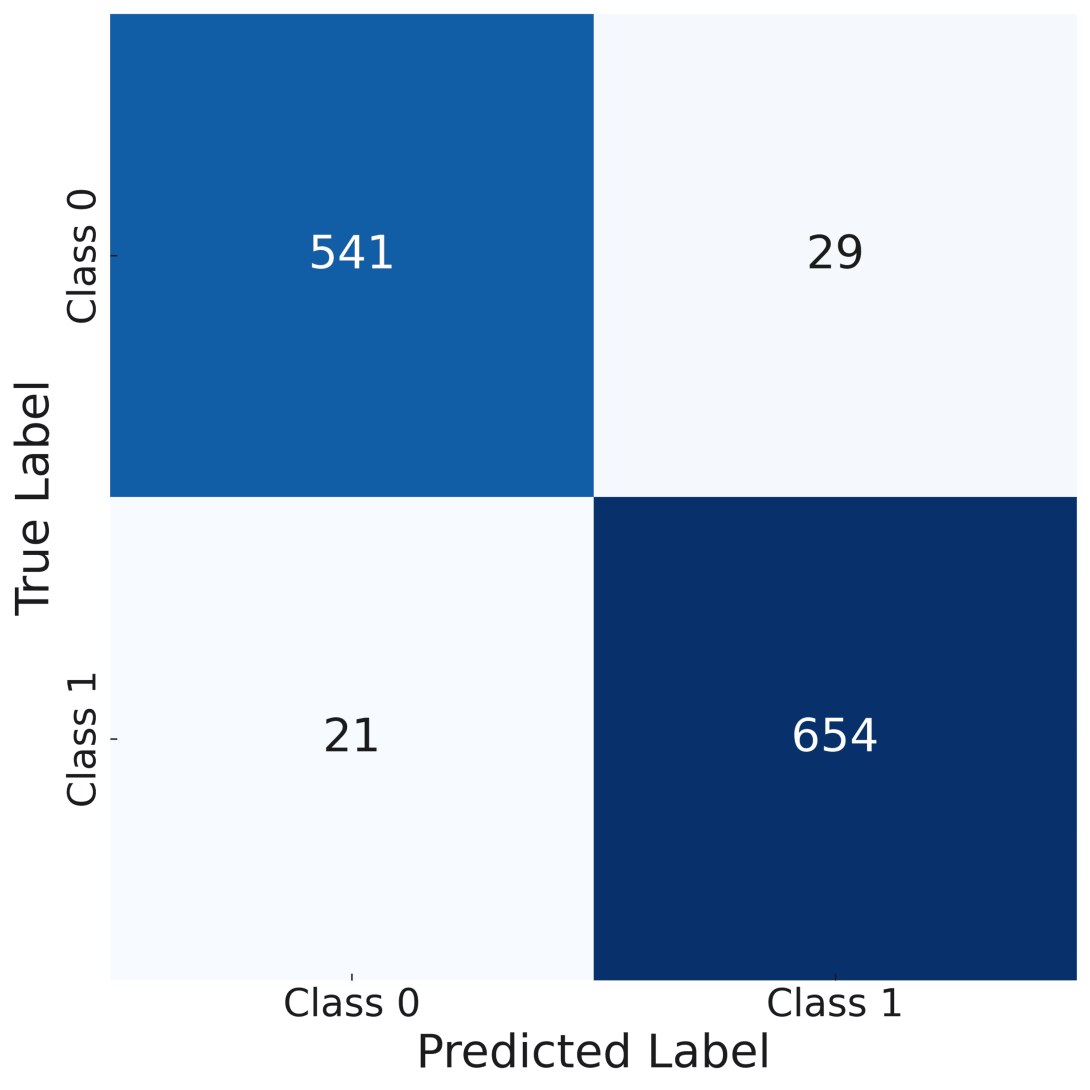
Confusion matrix for gender estimation model (model 1) Class 0 - Male Class 1 - Female

Through the confusion matrix shown in Figure 3, the model's performance in classification can be assessed. Each element of the confusion matrix represents the number of samples that belong to a certain class, and which are correctly or incorrectly classified by the model. On the diagonal elements of the confusion matrix, there are numbers that represent exactly classified samples for each class, while off the diagonal are the numbers that represent wrong classified samples. The matrix enables the analysis of different types of errors made by the model, such as what are false positive and false negative predictions.

Based on the values presented in the confusion matrix, it is possible to calculate model accuracy. Accuracy is the proportion of correct predictions and measures how well the classification predicts the condition. For the data given in the confusion matrix in Figure 3, the calculated accuracy is given by the following formula:

$accuracy=\frac{correct\;predictions}{all\;predictions}=\frac{541+654}{541+654+29+21}=0.9598$

The calculated accuracy of 0.9598 indicates that the model correctly classified 95.98% of the instances in the dataset, demonstrating a high level of performance in distinguishing between positive and negative cases. This suggests that the model is reliable for the given classification task.

Age estimation for individuals aged from 12 to 24 years

**Figure 4:**
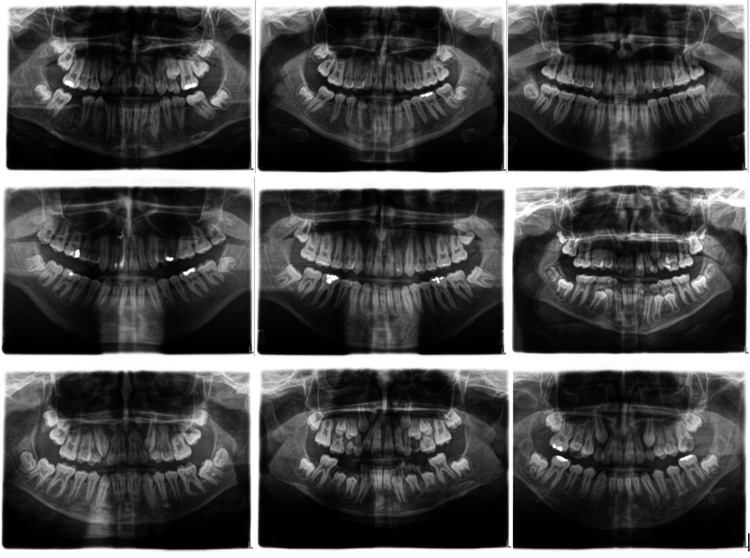
Sample of 9 images for the age estimation model from 12 to 24 years (model 2)

The second developed model focused on estimating individuals' ages from 12 to 24 based on 1193 orthopantomograms, employing a convolutional neural network. Figure 4 shows a random sample of nine out of 1193 orthopantomograms. An automated model created using a convolutional neural network for estimating the age of individuals between 12 and 24 years old employs the following approach for age categorization into three groups: the first group is under 16 years old, the second group ranges from 17 to 18 years old, and the third group is over 18 years old. For training the model, the majority of images were sourced from the database of a clinic for pediatric and preventive dentistry.

**Figure 5:**
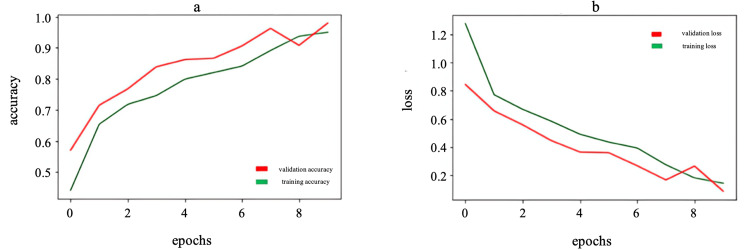
Accuracy (a) and loss (b) graphs for the age estimation model from 12 to 24 years (model 2)

Figure 5 shows that the accuracy increases and the loss decreases during training and validation, which indicates an improvement of the model without significant overfitting.

**Figure 6:**
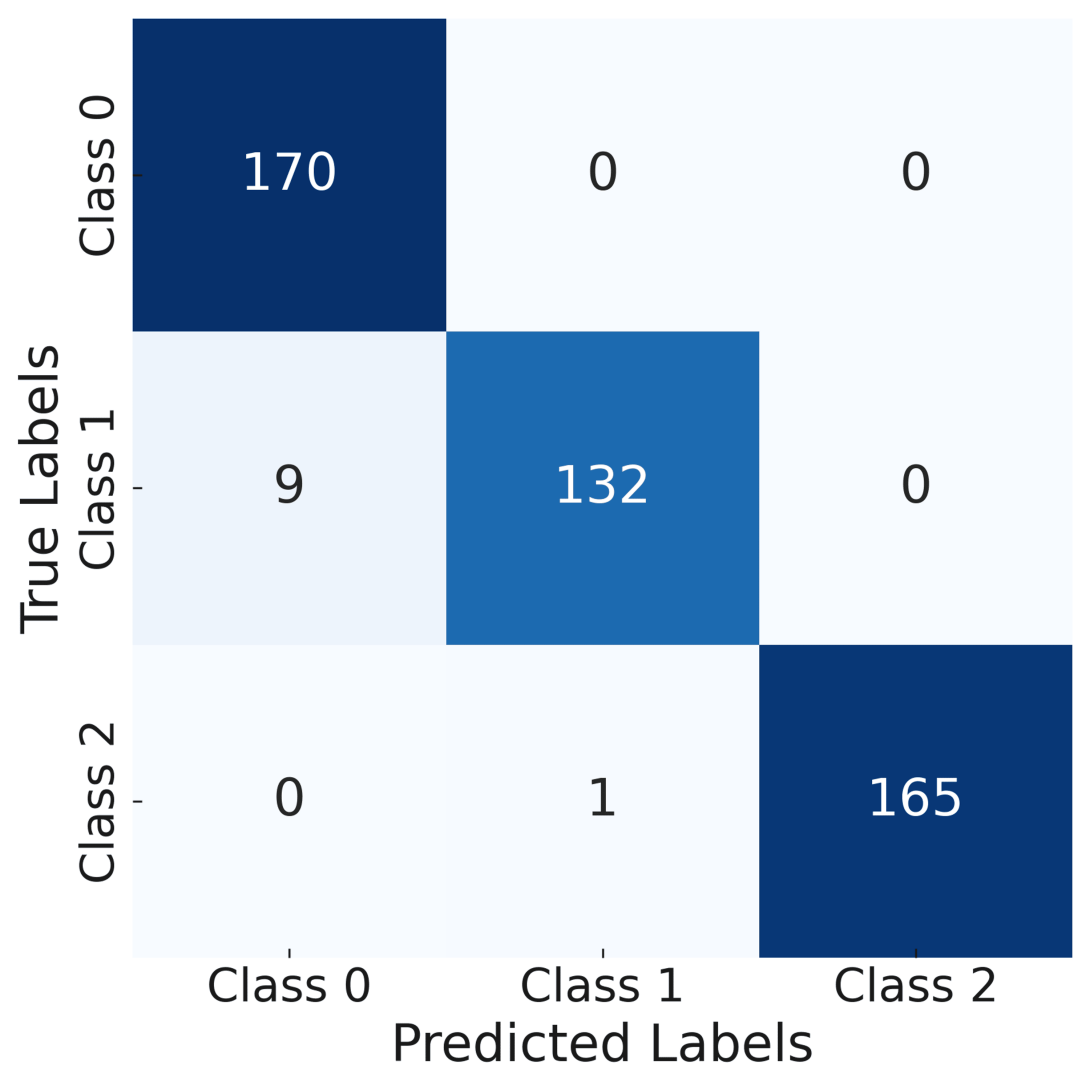
Confusion matrix for the age estimation model from 12 to 24 years (model 2) Class 0 - Age from 12 to 16 years Class 1 - Age from 17 to 18 years Class 2 - Age from 19 to 24 years

Through the confusion matrix shown in Figure 6, the accuracy of the model can be evaluated because on the diagonal there are numbers representing correctly classified samples for each class.

Based on the values presented in the confusion matrix model accuracy is calculated. For the data given in the confusion matrix in Figure 6, the calculated accuracy is given by the following formula:

$accuracy=\frac{correct\;predictions}{all\;predictions}=\frac{170+132+165}{170+9+132+1+165+4\cdot 0}=0.9790$

The calculated accuracy of 0.9790 shows that the model correctly classified 97.90% of the samples, indicating strong performance in differentiating among the three given classes. This suggests that the model is highly reliable for the intended classification task.

Age estimation for individuals aged from 20 to 70 years

**Figure 7:**
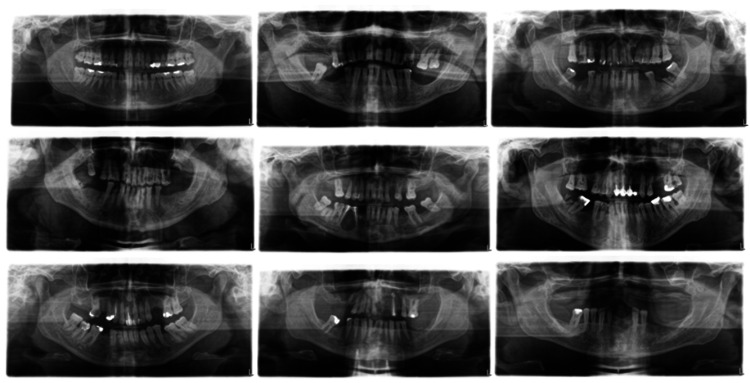
Sample of 9 images for the age estimation model from 20 to 70 years (model 3)

The third developed model focused on estimating individuals' ages from 20 to 70 based on 3020 orthopantomograms, employing a convolutional neural network. Figure 7 depicts a random sample of nine out of 3020 orthopantomograms. In the presented model, age estimation using a convolutional neural network is performed within the range of 20 to 70 years. The model is divided into five classes with intervals of 10 years between each class, enabling classification into different age groups. Input data includes images from all Clinics of the Faculty of Dentistry in Sarajevo because the range of age groups is large and present at each of the above.

**Figure 8:**
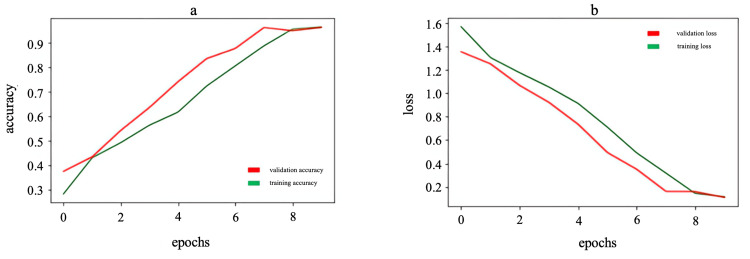
Accuracy (a) and loss (b) graphs for the age estimation model from 20 to 70 years (model 3)

Figure 8 shows that accuracy increases and loss decreases during training and validation. This is good because it indicates that the model is successfully learning from the data and getting better at classification, reducing errors in the process.

**Figure 9:**
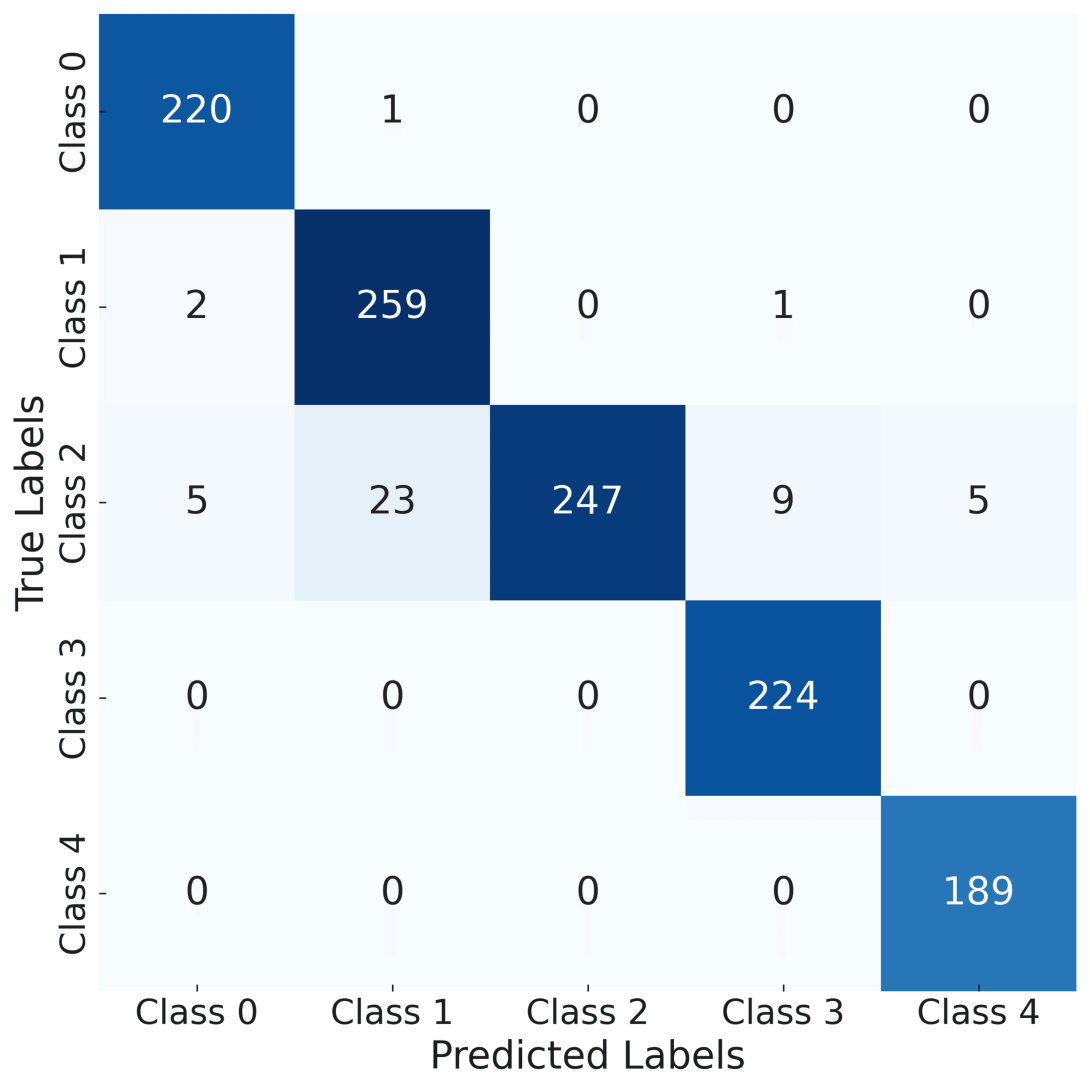
Confusion matrix for the age estimation model from 20 to 70 years (model 3) Class 0 - Age from 20 to 30 years Class 1 - Age from 31 to 40 years Class 2 - Age from 41 to 50 years Class 3 - Age from 51 to 60 years Class 4 - Age from 61 to 70 years

Through the confusion matrix shown in Figure 9, the accuracy of the model can be evaluated.

Based on the values presented in the confusion matrix, the accuracy for the third model is calculated. For the data given in the confusion matrix in Figure 9, the calculated accuracy is given by the following formula:

$accuracy=\frac{correct\;predictions}{all\;predictions}=\frac{220+259+247+224+189}{220+1+2+259+1+5+23+247+9+5+224+13\cdot 0}=0.9612$

The calculated accuracy of 0.9612 demonstrates that the model correctly classified 96.12% of the samples, highlighting its strong performance in distinguishing among the five given classes. This result suggests that the model is highly reliable for the intended classification task.

## Discussion

The developed CNN models for gender and age estimation based on dental X-ray images show that the usage of AI in forensic dentistry can be useful in identifying the individuals as it was shown in previous studies. The models developed in this research show how AI in forensic dentistry can provide useful insights for personal identification supporting the findings of previous research, such as [14], where high accuracy was achieved for gender estimation using dental X-rays. The use of a large number of high-quality data showed the importance of achieving higher accuracy for training and test sets which aligns with prior studies' conclusions that highlighted the importance of dataset reliability for developing models with high accuracy [15, 18]. The first model created to predict gender used 3, 716 orthopantomograms and attained an accuracy rate of 95.98%. This high level of accuracy showcases the model's ability to differentiate between male and female X-ray images effectively. The model's capacity to improve its accuracy and reduce loss over time suggests an ability to generalize without overfitting. These findings reinforce the idea that dental attributes possess gender-related features that deep learning models can capture successfully. High accuracy plays a role, in investigations as determining gender serves as an initial step in narrowing down possible matches. Furthermore, model 2 concentrated on predicting age within the 12 to 24-year range by classifying it into three groups: under 16 years old between 17 and 18 years old, and over 18 years old. Model 2 showed a high accuracy of 97.90% highlighting its usefulness in classifying individuals into age groups successfully. By distinguishing between these age ranges effectively Model 2 could be useful for age estimation in forensic dentistry where assessing dental age proves to be difficult due to the similarities in X-ray images among young individuals. Model 3 predicts the age of individuals from 20 to 70 years categorizing them into five 10-year intervals. The model showed a high accuracy of 96.12% categorizing individuals into their adequate age groups. The high accuracy reflects the model's capacity to capture differences in dental features that correspond to aging processes. Across all three models, by analyzing the training and validation accuracy it can be seen that there were no signs of overfitting, which proves that the models can be used in real-life scenarios. The accuracy values of 95.98%, 97.90%, and 96.12% for the gender estimation and age estimation models respectively, indicate high performance levels of the models, making these models useful tools for forensic identification tasks. These high accuracies were achieved because of the capability of CNNs to recognize hidden features in dental X-ray images by using convolutional layers. The high accuracy of model 1 supports the idea that dental features can be effectively recognized by AI and that AI methods can be successfully used in gender estimation as shown in studies [15] and [16]. These studies analyzed similar methods for gender estimation based on dental X-ray images. This research highlights the potential of the usage of CNNs in gender estimation in forensic dentistry. Models 2 and 3 that were developed in this study contribute to the existing knowledge by addressing the challenges in differentiating age groups, particularly in young individuals with overlapping dental features, as previously discussed in [18] and [19]. By using CNNs, developed models showed high accuracy in predicting the age of individuals. These models support previous research [20, 21] where AI is used for age estimation based on orthopantomograms in forensic dentistry. The low difference between the training and validation accuracy shows that models were not overfitted, from which can be concluded that these models could be used in real-world forensic applications on new unseen data which was shown in studies [19] and [20]. By estimating age with high accuracy these models can help in investigation processes. These models also align with the practical usage of gender and age estimation that was shown in [22] and [23], where AI methods were shown to improve the efficiency and accuracy of forensic identification. Automation of the process of identification of individuals based on orthopantomograms supports automation initiatives presented in [24] and [25]. The ability to extract complex features from dental X-rays using CNNs also complements the potential applications of AI in forensic odontology for person identification, as discussed in [26] and [27]. The development of these models has several practical implications for forensic dentistry. Gender estimation is often the first step in the identification of individuals. The ability to determine gender from dental X-rays with high accuracy is an important step of the investigative process. Age estimation is important for both identifying individuals and for legal issues where age-related and minors' rights are involved in the investigation process.

The limitation of this research is that images with visible bone fractures, deformities, injuries, loss of functional dentition, and/or dental implants and prosthetic restorations were excluded, which could be considered for future research.

## Conclusions

In this research, three CNN models were developed for gender and age estimation based on dental X-ray images. Determining gender and age is essential for the identification of individuals in forensic dentistry, and the usage of CNNs provides a new and innovative approach to determining gender and age. Developed CNN models had high accuracy showing the great potential of using CNNs in the age and gender in forensic dentistry based on dental X-rays. 

Model 1, which was developed for gender estimation, had an accuracy of 95.98%, showing the possibility of CNNs to differentiate between male and female individuals using dental orthopantomograms with high accuracy. Similarly, the two age estimation models-one for individuals aged 12 to 24 years and the other for individuals aged 20 to 70 years achieved accuracies of 97.90% and 96.12%, respectively. These results indicate the models’ strong performance in categorizing dental X-rays across distinct age groups, which is critical for forensic identification. The developed models showed high accuracies for training and validation set with no signs of overfitting indicating that the models could be used in real-life scenarios on new unseen data which is important in forensic dentistry. The models were trained on specific datasets and applied to specific populations from one geographical region, which may limit their usage to other populations from other geographical regions. Future work should focus on expanding and diversifying the dataset to include images from different populations enabling models to differentiate individuals across a wider demographic spectrum. Features, such as genetic markers or morphological characteristics of teeth could provide additional information for more precise gender and age determination. Images that were excluded for this research such as visible bone fractures, deformities, injuries, loss of functional dentition, and/or dental implants and prosthetic restorations could be considered for inclusion in future research.

The development of new methods for collecting dental traces and the application of methods such as three-dimensional scanning, could be used to improve the accuracy of gender and age estimation. The application of artificial intelligence and convolutional neural networks in forensic dentistry requires a high level of expertise and knowledge of specialists, therefore it is necessary to work on the development of new training programs for forensic dentists to improve their knowledge and skills in the application of new automated models for quick and accurate identity assessment, as well as understanding anthropological trends throughout history and evolution. This research supports the research of the potential of AI in forensic dentistry. Developed models show the effectiveness of CNN models in gender and age estimation. This research increases the role of AI methods for gender and age estimation in forensic fields.
